# Cloning and characterization of a novel 2-ketoisovalerate reductase from the beauvericin producer *Fusarium proliferatum* LF061

**DOI:** 10.1186/1472-6750-12-55

**Published:** 2012-08-23

**Authors:** Tao Zhang, XiaoPeng Jia, Ying Zhuo, Mei Liu, Hong Gao, JinTao Liu, Lixin Zhang

**Affiliations:** 1 Chinese Academy of Sciences Key Laboratory of Pathogenic Microbiology and Immunology, Institute of Microbiology, Chinese Academy of Sciences, Bei’er Tiao Road, Zhongguancun Haidian District, Beijing, 100190, China; 2 Graduate University of Chinese Academy of Sciences, Beijing, 100190, China

**Keywords:** 2-Kiv Reductase, Beauvericin, *Fusarium proliferatum* LF061

## Abstract

**Background:**

The ketoisovalerate reductase (EC 1.2.7.7 ) is required for the formation of beauvericin via the nonribosomal peptide synthetase biosynthetic pathway. It catalyzes the NADPH-specific reduction of ketoisovaleric acid to hydroxyisovalerate. However, little is known about the bioinformatics’ data about the 2-Kiv reductase in *Fusarium*. To date, heterologous production of the gene *KivRFp* from *Fusarium* has not been achieved.

**Results:**

The *KivRFp* gene was subcloned and expressed in *Escherichia coli* BL21 using the pET expression system. The gene *KivRFp* contained a 1,359 bp open reading frame (ORF) encoding a polypeptide of 452 amino acids with a molecular mass of 52 kDa. Sequence analysis indicated that it showed 61% and 52% amino acid identities to ketoisovalerate reductase from *Beauveria bassiana* ATCC 7159 (ACI30654) and *Metarhizium acridum* CQMa 102 (EFY89891), respectively; and several conserved regions were identified, including the putative nucleotide-binding signature site, GXGXXG, a catalytic triad (Glu405, Asn184, and Lys285). The KivRFp exhibited the highest activity at 35°C and pH 7.5 respectively, by reduction of ketoisovalerate. It also exhibited the high level of stability over wide temperature and pH spectra and in the presence of metal ions or detergents.

**Conclusions:**

A new ketoisovalerate reductase KivRFp was identified and characterized from the depsipeptide-producing fungus *F*. *proliferatum*. KivRFp has been shown to have useful properties, such as moderate thermal stability and broad pH optima, and may serve as the starting points for future protein engineering and directed evolution, towards the goal of developing efficient enzyme for downstream biotechnological applications.

## Background

**Figure 1:**
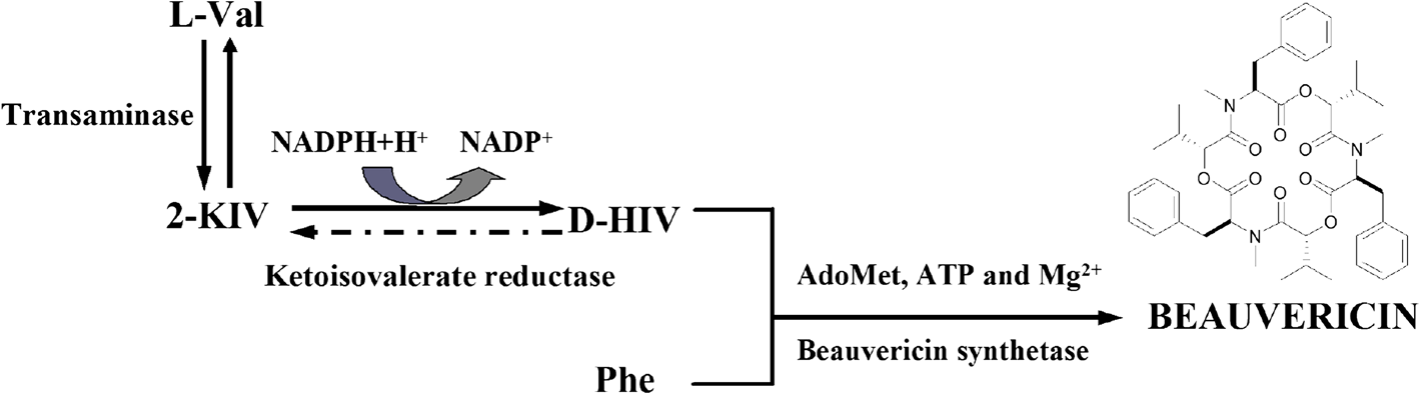
**Overall mechanism of beauvericin biosynthesis in *Fusarium *species.**

Cyclooligomer depsipeptides (CODs) are a prominent class of bioactive peptides produced from various Fungi by large multimeric enzyme complexes called nonribosomal peptide synthetases (NRPSs). There are many reports about CODs which contain various hydroxyl acids, such as D-hydroxyisovalerate (D-Hiv); examples include the beauvericin [1], enniatins [2], destruxin [3], bassianolide [4], fusafungine [5]. Beauvericin, produced by certain fungi of *Beauveria*[1], *Isaria*[6] and *Fusarium*[7] that exhibits insecticidal, displays antimicrobial, anti-tumor activities, reverses multidrug resistance in *Candida albicans*[8-11]. Its molecular structure consists of an alternating sequence of three *N*-methyl-L-phenylalanine and three D-hydroxyisovaleric acids (Figure 1) [12]. Thus, the hydroxyl acid is a key intermediate in the formation of the beauvericin.

In mammals, the catabolism of the branched-chain amino acids (BCAAs) leucine, isoleucine, and valine proceeds by two-step process, *i*.*e*. transamination step (aminotransfer) and oxidation step. BCAAs are usually transaminated to form 2-keto acids by branched-chain amino acid transaminases (BCATs). These 2-keto acids are oxidatively decarboxylated by mitochondrial branched-chain 2-ketoacid dehydrogenase [13-15] and converted to acyl CoA. However, in some fungal species, namely the hydroxysiovalerate-containing CODs producers, there is another pathway leading from L-Val to D-Hiv via the keto acid (Figure 1). D-hydroxyisovalerate dehydrogenase, catalyzes the reversible reduction of 2-ketoisovalertate (2-Kiv), the intermediate of branched-chain amino acid metabolism [16], to D-Hiv in the presence of NADPH, which cannot be replaced by NADH [17-19]. So far, D-HivDH has been purified and partially characterized exclusively from the enniatin producer, *Fusarium sambucinum*[17,19], but the corresponding protein and gene sequences were not determined. Xu *et al*., demonstrated the novel *kivr*-encoding 2-ketoisovalerate reductase is the sole supplier of D-Hiv for both beauvericin and bassianolide biosynthesis in *Beauveria bassiana* ATCC 7159 [20].

On the other hand, little is known about the bioinformatics’ data about the 2-Kiv reductase (2-KivR) in *Fusarium*, thus warranting further study. To date, heterologous production of the gene *KivRFp* from *Fusarium* has not been achieved; similarly, very little information is available about the role of the KivRFp in the regulation of beauvericin biosynthesis. In order to get a closer insight into the unique and intriguing features of the ketoisovalerate reductase in the *Fusarium* genus, we undertook both genetic and biochemical studies. In the present article, we describe the identification and functional analysis of the *KivRFp* of *Fusarium proliferatum* LF061. Our report demonstrates that the KivRFp constituted a new member of family ketonate oxidoreductases.

## Methods

### Chemicals

Chemicals were of the highest purity commercially available. Pyruvate, 2-Kiv, 2-ketovalerate, 2-ketoglutarate and 2-ketobutyrate were obtained from TCI Japan Ltd. D-Hiv were purchased from Sigma. NADH, NADPH, NADP^+^ were products of Roche.

### Bacterial strains, plasmids, and culture

The starting strains and plasmids used in this study are listed in Table 1. *E. coli* was grown at 37°C in Luria–Bertani (LB) medium supplemented with appropriate antibiotics [21]. When required, ampicillin was added at a final concentration of 100 μg/mL, kanamycin at 25 μg/mL, and chloramphenicol, at 12.5 μg/mL. *Fusarium proliferatum* LF061 (deposited in China General Microbiological Culture Collection under the accession number of CGMCC 3.1777) was maintained on potato dextrose agar (PDA).

**Table 1:** Starting bacterial strains and plasmids used in this study

**Strain or plasmid**	**Description**	**Source**
**Strains**		
*E. coli* TOP10	*lac*х*74 recA1 deoR F – mcrA* Δ (*mrr-hsdRMS-mcrBC*) *ϕ80 lacZ*Δ*M15*Δ *araD139*Δ *(ara-leu)7697 galU galK*	Transgen
*E. coli* BL21(DE3)	*F–, ompT, hsdSB (rB–, mB–), dcm, gal, λ(DE3), pLysS, Cmr*	Novagen
*Fusarium proliferatum* LF061	Wild strain, producing beauvericin isolated from marine sample	This study
*E*. *coli* TOP10-*KivRFp*	Positive clone of artificial gene synthesis, which carries the *KivRFp* gene fragment	This study
*E. coli* BL21(DE3)-*KivRFp*	Positive clone, which carries the p*KivRFp*-His expression vector	This study
**Plasmids**		
pUCE	Cloning vector; Ap^r^	Inovogen
pET28a	Expression vector; Km^r^	Novagen
pUCE-*KivRFp*	pUCE, which carries the complete ketoisovalerate reductase (*KivRFp*)	This study
p*KivRFp*-His	pET28a carrying amplified *EcoR*I-*Nde*I fragment containing ketoisovalerate reductase gene (*KivRFp*)	This study

Ap^r^, ampicillin resistant; Km^r^, kanamycin resistant.

### DNA preparation and manipulation

*E. coli* cells were transformed by the calcium chloride procedure [21]. Recombinant plasmid DNA was isolated by the method of Birnboim and Doly [22]. For sequencing, this DNA was further purified by polyethylene glycol precipitation [21]. Restriction enzymes, T4 DNA ligase and calf intestinal alkaline phosphatase were purchased from New England Biolabs (Ipswich, USA) or Takara (Tokyo, Japan) and used according to the manufacturers’ instructions. BugBuster Ni-NTA His. Bind Purification Kit was purchased from Novagen (Code No. NV70751-3, Novagen).

### Phylogenetic analysis

Deduced amino acid sequences of 13 ketonate reductases or homologous proteins were subjected to protein phylogenetic analysis. A phylogenetic tree was generated using the neighbor joining method of Saitou and Nei [23] with MEGA 4.0 software [24]. A total of 4 sequences were aligned with the CLUSTAL_W program [25] and visually examined with BoxShade Server program. The length of each branch pair represents the evolutionary distance between the sequences.

### Heterologous expression of gene *KivRFp* and purification of recombinant KivRFp

Artificial gene synthesis was finished by Innovogen Tech. Co. (Beijing, China). Jcat (Java Codon Adaptation Tool) [26] and OPTIMIZER [27] were used to improve heterologous protein production. The integrity of the nucleotide sequence of all newly constructed plasmids was confirmed by DNA sequencing. The primer pairs with restriction enzyme sites for *Hin*dIII and *Nde*I were designed to generate an N-terminal His-tag of the recombinant ketonate reductase. The modified *KivRFp* gene was cloned into an expression vector, pET28a (+) and the recombinant plasmid p*KivRFp*-His was transformed into *E. coli* BL21 (DE3) cells. When the cell density at 600 nm reached around 0.6, expression of recombinant KivRFp protein was initiated by addition of 0.6 mM isopropylthio-*β*-D-galactoside and continued cultivation for additional 6 h at 20°C. Cells were harvested by centrifugation at 6,000 × g for 5 min, washed twice with ice-cold 50 mM sodium phosphate buffer (pH 8.0) and resuspended in the same buffer containing 10 mM imidazole, disrupted by sonification in an ice-water bath (60 times, 5 s). Recombinant KivRFp reductase was applied to metal-chelating chromatography using Ni-NTA affinity chromatography (Novagen) according to the manufacturer’s instructions.

Polyacrylamide gel electrophoresis of enzyme in the presence of sodium dodecyl sulfate (SDS) was carried out by the method of Sambrook and Russell [21]. Protein concentrations were determined by a modified Bradford procedure with bovine serum albumin as a standard [28].

### Enzyme assays

The standard 2-Kiv reductase assay mixture contained 50 mM sodium phosphate buffer (pH 7.0), 0.7 mM 2-Kiv, 0.29 mM NADPH, and enzyme in a final volume of 3.0 mL. The reaction was initiated by the addition of substrate, and the decrease in absorbance at 340 nm (A_340_) was measured at 35°C, which was performed using an UNICO 2802 UV/VIS spectrophotometer. A molar extinction coefficient of 6.22 cm^2^/pmol NADPH was used for the calculation of enzyme activity. One unit was defined as the amount of enzyme caused the oxidation of 1 μmol of NADPH per min. The reaction mixture for the assay of the reverse reaction contained 50 mM sodium phosphate buffer (pH 8.0), 2.8 mM NADP^+^, 5.7 mM D-Hiv, and enzyme in a final volume of 3.0 mL. The increase in the rate of reduction of NADP^+^ due to oxidation of D-Hiv was measured at 340 nm (45°C) [18,19,29].

### Characterization of recombinant **KivRFp** and biochemical properties

The purified KivRFp was subjected to a series of biochemical analysis, including determine the pH optimum, temperature optimum, and effects of various detergents and metal ions. All measurements were carried out in triplicate. The values were the mean of the data. The substrate specificity of the purified KivRFp protein was performed using the following substrates: pyruvate, 2-Kiv, 2-ketovalerate, 2-ketobutyrate, 2-ketocapronate and 2-ketoglutarate.

The optimum temperature of purified KivRFp was determined by assaying reductase activities in a 50 mM sodium phosphate buffer (pH 8.0) for a temperature range of 25-65°C, in which 2-Kiv (0.7 mM) acted as substrate. Optimal pH was determined by examining the activity of the enzyme after incubation at 40°C for 10 min using 2-Kiv (0.7 mM) as substrate. The buffers used were: 50 mM sodium phosphate buffer (pH 5.0-8.0), 50 mM Tris–HCl buffer (pH 8.0-10.5).

Various metal ions (CoCl_2_, CaCl_2_, ZnCl_2_, MgCl_2_, K_2_SO_4_, FeSO_4_, CuCl_2_, MnCl_2_, and FeCl_3_), and chelating agent EDTA at final concentrations of 5 mM were added to the enzyme in 50 mM sodium phosphate buffer (pH 8.0), that was assayed for 2-Kiv reductase activity following preincubation at 40°C. Effect of detergents on reductase activity was determined by incubating the enzyme for 10 min at 40°C in 50 mM sodium phosphate buffer (pH 8.0), containing Tween-20, Tween-80, Triton X-100, *β*-mercaptoethanol, sodium dodecyl sulfate (SDS), cetyltrimethyl ammonium bromide (CTAB), diethylpyrocarbonate (DEPC), phenylmethanesulfonyl fluoride (PMSF), dimethyl sulfoxide (DMSO). The concentrations of metal ions, EDTA, detergents, and surfactants used were 5 mM, 3 mM, and 0.5% (v/v), respectively. The activity of the enzyme preparation in the absence of metal ions and detergents before incubation was defined as the 100% level.

### Nucleotide sequence accession number

The DNA sequence of KivRFp from *Fusarium proliferatum* LF061 was deposited in GenBank under accession number of JQ922252.

## Results

### Cloning and sequence analysis of KivRFp

We have recently cloned and functionally characterized beauvericin biosynthetic locus (*bea*) of the *F*. *proliferatum* LF061 genome (unpublished data). The *FpBEAS* gene encoding the beauvericin synthetase was found to be clustered with a gene (*KivRFp*) encoding a deduced putative 2-ketoisovalerate reductase (KivRFp). Amino acid sequence alignment indicated that KivRFp exhibited low identity with other ketopantoate reductases or hypothetical proteins in GenBank. KivRFp shared the highest sequence identity with the KIVR from *B*. *bassiana* ATCC 7159 (ACI30654, 61% identity/75% similarity), followed by the putative 2-ketopantoate reductase from *Metarhizium acridum* CQMa 102 (EFY89891, 56% identity/70% similarity). Similarity was also detected with the hypothetical or putative 2-dehydropantoate reductases from *Gibberella zeae* PH-1 (anamorph: *F*. *graminearum*) and *F*. *oxysporum* Fo5176 (EGU84839, 49% identity; and EGU75687, 47% identity, respectively), and also with functionally uncharacterized terminal reductase domain of the *Xylaria* sp. bassianolide synthetase (ABR28366, 30% identity/48% similarity).

**Figure 2:**
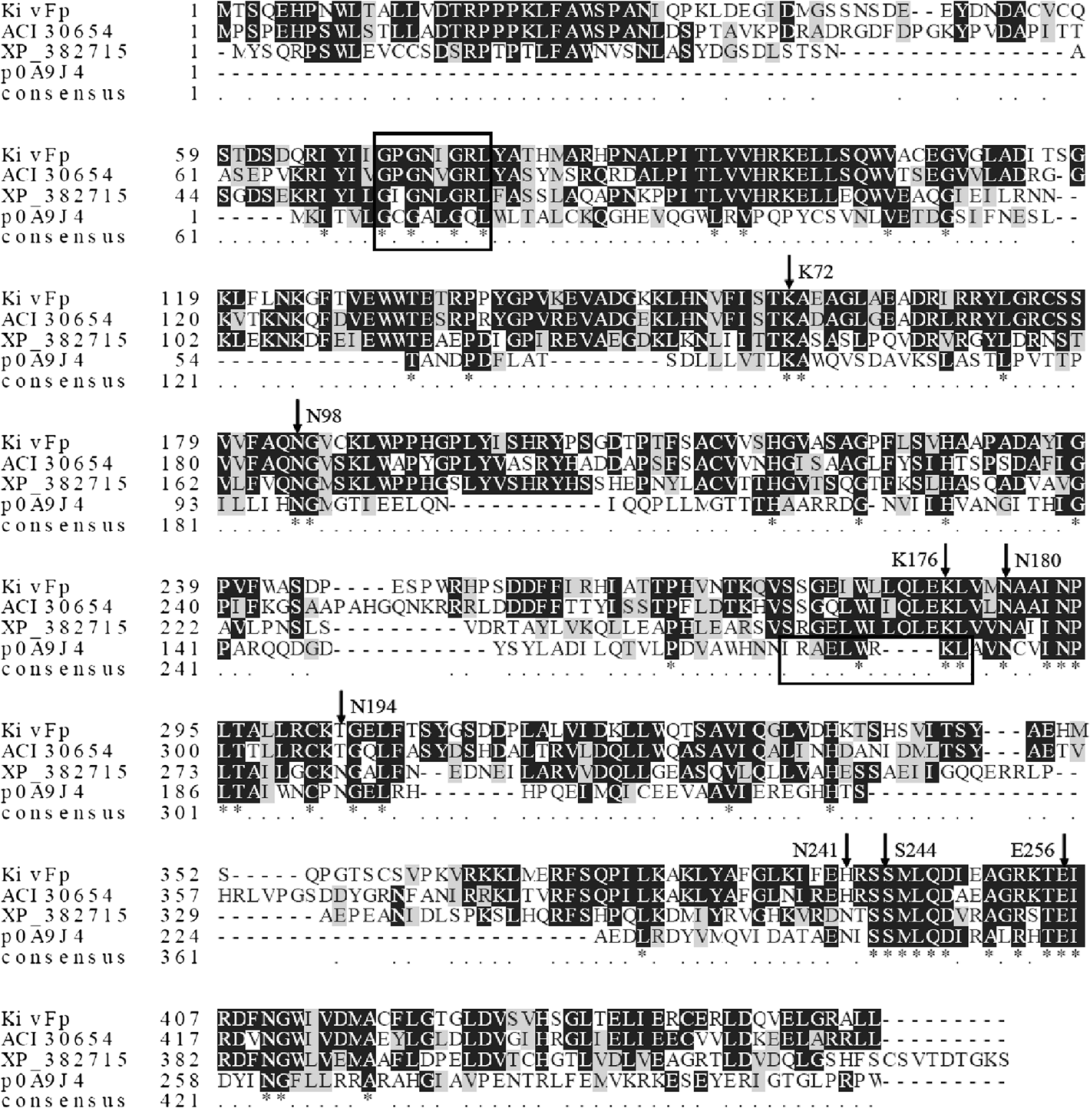
**Conserved sequence blocks from multiple sequence alignment of KivRFp from *Fusarium proliferatum *LF061 and other related proteins.** Sequences alignment was carried out with CLUSTALW [25] and BoxShade Server ( http://www.ch.embnet.org/software/BOX_form.html). XP_382715, hypothetical protein from *Gibberella zeae* PH-1; ACI30654, ketoisovalerate reductase from *Beauveria bassiana* ATCC 7159; P0A9J4, PanE ketopantoate reductase from *E*. *coli*. The alignment was optimized and annotated with secondary structure predictions in SWISS-MODEL [32] by use of the determined crystal structure of PanE (2OFP, chain A). Identical amino acids are shown on black ground. Key amino acid residues mentioned in the text are labeled with arrows and numbered with reference to the PanE sequence. The nucleotide binding signatures and the hinge region separating the N-terminal Rossmann fold and the C-terminal catalytic domain in PanE are boxed [30,33].

**Figure 3:**
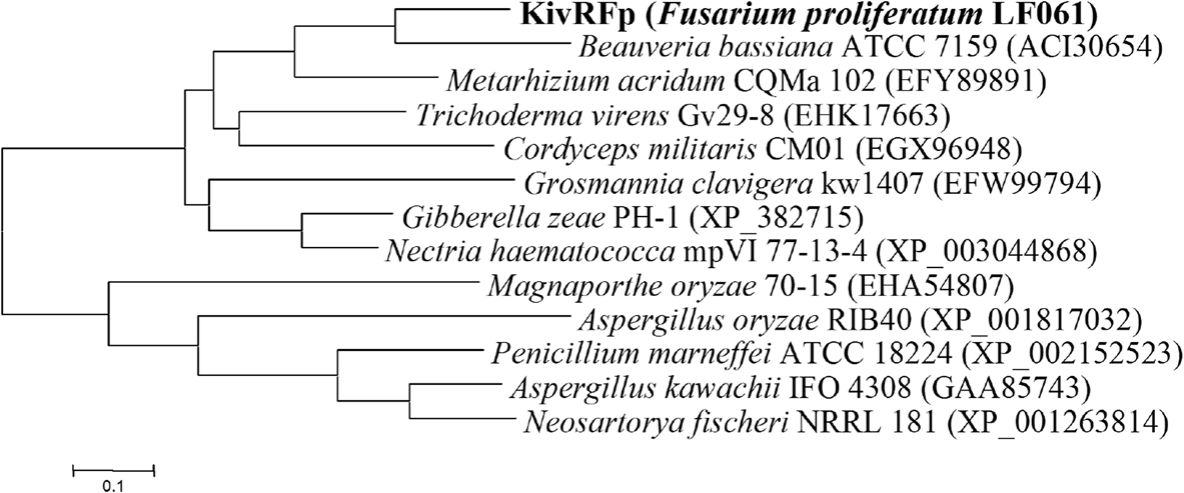
**Phylogenetic analysis of KivRFp and closely related proteins.** Phylogenetic analysis was performed using the program MEGA4.0 [23,24]. Except for KivRFp, the protein sequences for dehydrogenase were retrieved from GenBank ( http://www.ncbi.nlm.nih.gov). The numbers at node indicate the bootstrap percentages of 1000 resamples.

Various ketonate reductases contain the conserved GXGXXG nucleotide-binding signature. And also, a conserved glutamate (Glu), an asparagines (Asn) and a lysine (Lys), together constituting a catalytic triad [30,31]. The amino acid sequence alignment to other ketonate reductases retrieved from GenBank, identified the conserved motifs, including the putative GXGXXG nucleotide-binding signature and a Glu-Asn-Lys triad active site architecture (Figure 2). Thus, KivRFp probably uses a catalytic triad consisting of the glutamate (Glu405), the asparagine (Asn184) and the highly conserved lysine (Lys285) for catalysis. All these residues are conserved in the *F*. *proliferatum* KivRFp that might contribute to the formation of the active hole and involve directly in the catalytic process [30,31]. Furthermore, to clarify the phylogenetic relationship of the KivRFp with other ketonate reductases, a neighbor joining phylogenetic tree was constructed using the amino acid sequence of the ketonate reductive enzymes. As shown in Figure 3. In this tree, KivRFp formed a distinct group with the KIVR of *B*. *bassiana* ATCC7159 (ACI30654), which is located closest to the branch of putative 2-ketopantoate reductase of *Metarhizium acridum* CQMa 102 (EFY89891), hypothetical protein of *Trichoderma virens* Gv29-8 and *Cordyceps militaris* CM01 (accession number EHK17663 and EGX96948 respectively). Moreover, KivRFp showed sequence and the structural similarity with ketopantoate reductases [32,33], but not with D-lactose or D-hydroxyisocaproate dehydrogenase [20]. These results suggest that the KivRFp is a new member of family ketonate reductases.

### Expression and purification of recombinant KivRFp

**Figure 4:**
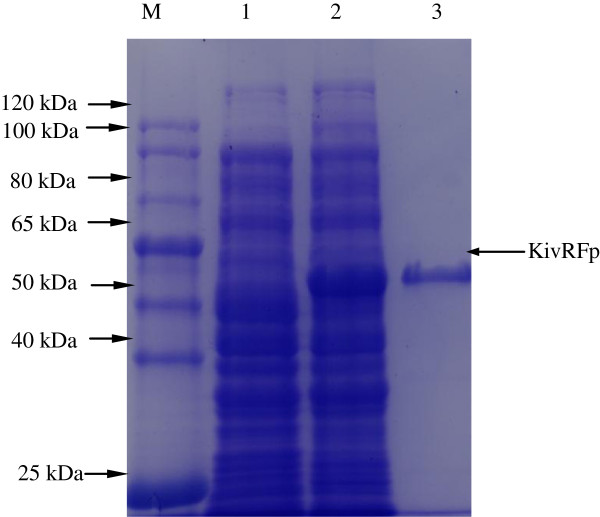
**SDS-polyacrylamide gel of overexpressed ketoisovalerate reductase KivRFp in *E. coli *.** Lane M: molecular weight protein marker (TransGen, Cat. No: DR301); lane 1, induced culture of *E. coli*/pET28a: total protein extract, as negative control; lane 2, total protein extract, induced culture of *E. coli*/ pKivRFp-His; lane 3: purified KivRFp (52 kDa).

To investigate the property of this KivRFp, optimized *KivRFp* gene was expressed as an N-terminal His-tag fusion protein using pET-28a (+) expression system in *E. coli* BL21(DE3). The recombinant protein was analyzed by SDS-PAGE and Coomassie brilliant blue staining (Figure 4). These results indicate that recombinant KivRFp protein is expressed (Mw, about 52 kDa), as which correlated well to the predicted full length of KivRFp. The purity of the purified protein was more than 98% according to SDS–PAGE analysis.

### Substrate and cofactor specificity of KivRFp

**Figure 5:**
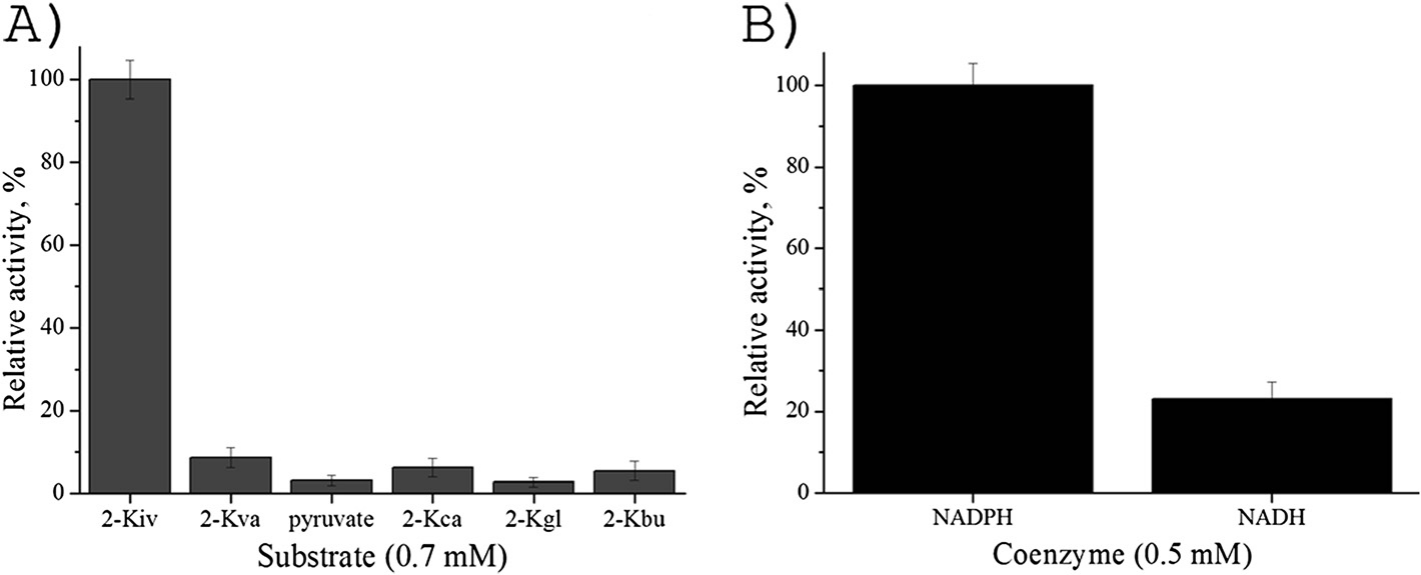
**Substrate specificity of overexpressed and purified ketoisovalerate reductase KivRFp.** 5**A**). Specific activity of reductive reaction of different homologous compounds using NADPH (0.29 mM) as coenzyme. 2-ketoisovalerate (2-Kiv), 2-ketovalerate (2-Kva), pyruvate, 2-ketocapronate (2-Kca), 2-ketoglutarate (2-Kgl), and 2-ketobutyrate (2-Kbu). 5**B**). Specific activity of reductive reaction of different coenzymes using 2-Kiv (0.7 mM) as substrate. Relative activity was shown as the percentage of the activity of the activity towards 2-ketoisovalerate. All measurements were performed in triplicate.

We expressed KivRFp as a hexahistidine-tagged (His-tagged) protein and investigated its substrate specificity. The enzyme exhibited the highest activity towards 2-Kiv as substrate, while the homologues compounds 2-ketovalerate, pyruvate, 2-ketocapronate, 2-ketoglutarate and 2-ketobutyrate were shown to be poor substrates (Figure 5A). And also, the KivRFp showed higher coenzyme specificity with respect to NADPH than NADH (Figure 5B), which is a little different from highly specific D-hydroxyisovalerate dehydrogenase from enniatin producer *F*. *sambucinum*.

### Effect of temperature and pH on KivRFp

**Figure 6:**
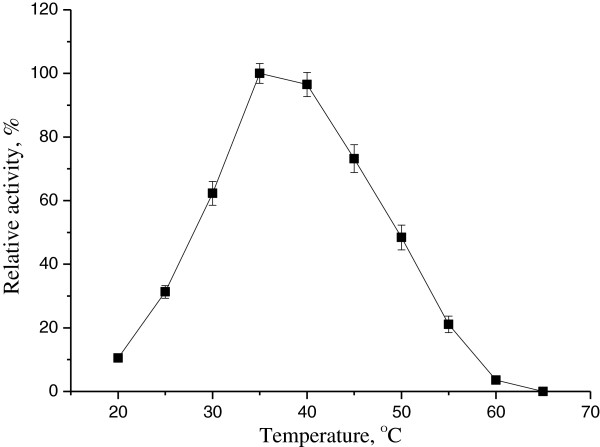
**Apparent temperature optimum of ketoisovalerate reductase KivRFp.** Relative activity of ketoisovalerate reduction at different temperatures by purified KivRFp. The activity was determined at different temperatures at pH 8.0 in 50 mM sodium phosphate buffer. The activity at 35°C was set as 100%. All measurements were performed in triplicate.

**Figure 7:**
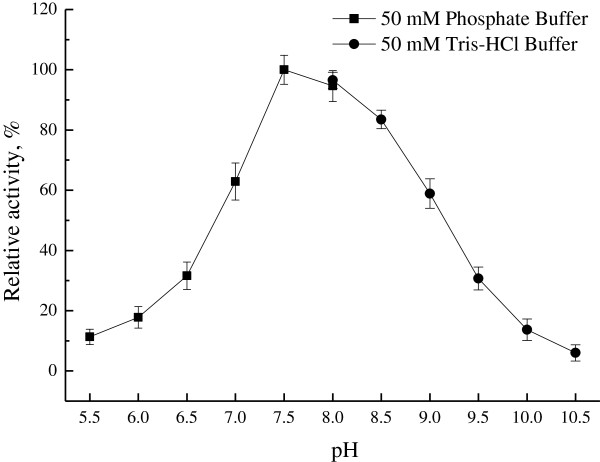
**Effect of pH on the purified ketoisovalerate reductase KivRFp.** Relative activity of ketoisovalerate reduction was performed in various pH buffers at 35°C (pH 5.0-8.0, 50 mM sodium phosphate buffer; pH 8.0-10.5, 50 mM Tris–HCl buffer). The activity at pH 7.5 was set as 100%. All measurements were performed in triplicate.

Reduction of 2-Kiv activity of KivRFp was determined from 25°C to 65°C. The purified KivRFp showed highest activity at 35°C. However, the activity of KivRFp reduced quickly above 55°C (Figure 6). Thermostability analysis showed that KivRFp was not stable and lost activity in less than 10 min at 60°C (data not shown). And also, the reductase showed activity in a rather broader pH range of 6.5-9.5. Maximal activity was observed at pH 7.5 and lost activity at pH 10.5 using 2-ketoisovalerate and NADPH as substrates (Figure 7). However, the optimum temperature and pH of oxidative reaction were found to be 40°C and 8.5 respectively (not shown).

### Effect of metal ions on KivRFp

The effects of metal ions and ethylenediamine tetraacetic acid (EDTA) on the KivRFp reductase activity were investigated by measuring the residual enzyme activity in their presence and depicted in Table 2. Among metal ions tested, the reductase activity was increased by Mg^2+^ (195%), Ca^2+^ (169%) and a slight increase observed by K^+^ (123%). Furthermore, the reduction activity was inhibited by Fe^2+^ and Co^2+^, moreover, almost totally inhibited by Cu^2+^, Mn^2+^, Zn_,_^2+^ and Fe^3+^ ( from 3% to 9% residual activity respectively), while the chelating agent EDTA had little effect, suggesting Mg^2+^ and Ca^2+^ are not determining factors, though they could promote the reductase enzyme activity.

**Table 2:** Effect of metal ions on esterase activity

**Compounds**	**Concentration (mM)**	**Relative activity (%)**
Control	0	100.0 ± 1.7
CoCl_2_	5	16.4 ± 2.9
K_2_SO_4_	5	123.2 ± 3.4
FeSO_4_	5	4.9 ± 1.6
CuCl_2_	5	7.8 ± 2.7
MnCl_2_	5	6.2 ± 1.3
EDTA	5	87.7 ± 3.2
FeCl_3_	5	7.9 ± 3.4
CaCl_2_	5	169.1 ± 3.7
ZnCl_2_	5	3.7 ± 1.8
MgCl_2_	5	195.6 ± 2.7

Activity without metal ions was set as 100%. All measurements were repeated three times.

### Effect of detergents and enzyme inhibitors on KivRFp

The effects of detergents and enzyme inhibitors on ketoisovalerate reductase (KIVR) reduction activity are shown in Table 3. A significant increase in reductive activity was observed with addition of 3 mM DTT (127%), Tween-80 (176%), DEPC (114%), and Tween-20 (129%), after 0.5 h preincubation with detergents at 37°C, which might help the substrate interaction with the catalytic triad. Moreover, 0.5% *β*-mercaptoethanol did not affect the reductive activity (102%), whereas CTAB, PMSF, Triton X-100, and SDS strongly inhibited enzymatic activity of KivRFp. The activity inhibition of KivRFp by PMSF suggests that serine is involved in the catalytic center, since PMSF could mimic the first transition state in ester bond hydrolysis presumably by linking to the hydroxyl group of serine in the active site covalently. The enzymatic activity of KivRFp is totally inhibited by ionic detergent and the probable reasons for these are ionic detergents such as SDS inhibits both inter- and intra-molecular protein-protein interaction.

**Table 3:** Effect of detergents and enzyme inhibitors on esterase activity

**Compounds**	**Concentration**	**Relative activity (%)**
Control	0	100.0 ± 2.1
DTT	3 mM	126.9 ± 4.9
CTAB	3 mM	42.7 ± 2.2
DEPC	3 mM	113.6 ± 2.7
PMSF	3 mM	53.6 ± 4.1
SDS	3 mM	6.3 ± 1.9
*β*-mercaptoethanol	0.5%	101.7 ± 2.6
Triton X-100	0.5%	60.8 ± 4.6
Tween-80	0.5%	176.4 ± 2.1
Tween-20	0.5%	128.7 ± 3.3

Activity without detergents and enzyme inhibitors was set as 100%. All measurements were repeated three times.

## Discussion

Ketoisovalerate reductases are types of oxidoreductases which are widely distributed from prokaryotes to eukaryotes and which are involved in primary and second metabolism [17,20,34-36]. 2-hydroxyisovalerate is a common 2-hydroxycarboxylate constituent of depsipeptides. Reduction of Kiv in fungi provides Hiv, a precursor for the biosynthesis of nonribosomal depsipeptides and aroma compounds. Fungal cyclooligomer depsipeptides synthetase A domains incorporate free 2-hydroxycarboxylate precursors for the synthesis of CODs, preformed by dissociated, independent, monofunctional, NADPH-dependent enzymes with high chiral specificity, exemplified by the D-Hiv dehydrogenase purified from the enniatin producer *F*. *sambucinum* (teleomorph: *G*. *pulicaris*) [17,19]. In contrast, known bacterial depsipeptide synthetases employ A domains that activate and load 2-ketocarboxylates as *β*-ketoacyl thioesters onto their cognate T domains, and utilize integrated ketoacyl reductase domains for the synthesis of 2-hydroxycarboxylate thioesters. This might suggest the biosynthesis of 2-hydroxycarboxylate precursors in bacteria and fungi may represent example of convergent evolution [20]. This enzyme, which plays a key role in beauvericin biosynthesis, catalyzes the reversible reaction of 2-Kiv to D-Hiv using NAD(P)H as a cofactor. 2-ketoisovalerate reductase differs from other NADPH-dependent oxidoreductases with broad substrate specificity by its high affinity for 2-Kiv (Figure 5A). The high specificity of KivRFp may also explain the fact that D-Hiv is the exclusive hydroxy acid component in beauvericin isolated from *Fusaria* (Logrieco A et al., 1998). To our knowledge this is the first report about the heterologous production of a ketoisovalerate reductase with higher substrate specificity for 2-Kiv.

KivRFp, which was cloned, expressed, and purified, was subjected to molecular and biochemical experiments in this study. The *F*. *proliferatum* KivRFp exhibits high amino acid sequence similarity to *B*. *bassiana* KIVR and deduced dehydropantoate reductases of fungi, together with low but significant sequence similarity predicted secondary structure to prokaryotic ketopantoate reductases such as PanE of *E*. *coli*[20,30,31,36]. KivRFp and the related *B*. *bassiana* KIVR are predicted to share the N-terminal *αβ*-Rossmann fold and the C-terminal *α*-helical catalytic domain architecture of the *E*. *coli* ketopantoate reductase. In the amino acid sequence of KivRFp, the conserved catalytic triad (Glu-Asn-Lys) and substrates stabilization active amino acids of ketonate oxidoreductases are still the same. In ketopantoate reductase PanE from *E*. *coli*, the protonated Lys176 (KivRFp: Lys285, Figure 2) could function as a general acid to polarize the carbonyl in the direction of ketopantoate reduction, facilitates the hydride transfer from NADPH to the C2 carbonyl of ketopantoate, and protonates the intermediate alkoxide [20,31]. Asn98 (KivRFp: Asn184) forms hydrogen binding both with the carboxylate of the ketopantoate and with the ribose of the nicotinamide: this orients the cofactor. And also, Glu256 (KivRFp: Glu405) and Lys72 (KivRFp: Lys158) further participate substrate binding to the NADPH [20]. Further stabilization is provided by a hydrogen bond between the Ser244 (KivRFp: Ser393) and carboxyl group of the ketopantoate, and between Asn180 (KivRFp: Asn289) and the *a*-hydroxy group of the product. And also, KivRFp showed sequence and the structural similarity with ketopantoate reductases, but not with D-lactose or D-hydroxyisocaproate dehydrogenase [20]. Further biochemical assay demonstrated that *Fusarium* KivRFp could utilize NADPH and NADH as coenzyme, while the NADPH is preferred (Figure 5B). In accordance to our results, bacterial chiral enzymes belonging to the D-ketoacid reductases superfamily also could utilize NADH as coenzyme, and also feature a conserved Arg-(Asp/Glu)-His active site triad [20,37]. Based on the phylogenetic, molecular, and biochemical analysis results, we propose that KivRFp is a new member of family ketonate oxidoreductases. Future work will establish the structure of this enzyme to gain more information about its catalytic mechanism.

## Conclusion

In conclusion, we identified a new ketoisovalerate reductase KivRFp from the depsipeptide-producing fungus *F*. *proliferatum*. KivRFp is expected to show high potential for downstream biotechnological applications including synthetic organic chemistry and combinatorial biosynthesis beauvericin homologues. This was confirmed by its extensive biochemical characterization, which revealed the enzymes substrate specificity, wide pH and temperature spectra, and also, stability towards addictives including metal ions and detergents. Enlargement of the ketoisovalerate reductase pool can be an immediate source of genetic modification, or yield enzymes that can be further specialized by directed evolution, and also, this would optimize their industrial applications.

## Abbreviation

BEA: Beauvericin; CODs: Cyclooligomer depsipeptides; NRPS: Nonribosomal peptide synthetases; D-Hiv: D-hydroxyisovalerate; 2-Kiv: 2-ketoisovalerate; CODS: Cyclooligomer depsipeptides synthetase; D-HivDH: D-Hiv dehydrogenase; 2-KivR: 2-Kiv reductase.

## Competing interests

The authors declare that they have no competing interests.

## Authors’ contributions

TZ participated in the design of experiments, and carried out the study and drafted the manuscript. XPJ, YZ, ML, HG, JTL and LXZ participated in its design and coordination and helped to draft the manuscript. All authors read and approved the final manuscript.

## Authors’ information

TZ, XPJ, YZ, ML, HG and LXZ, Chinese Academy of Sciences Key Laboratory of Pathogenic Microbiology and Immunology, Institute of Microbiology, Chinese Academy of Sciences, Beijing 100190, China. JTL, deceased.
